# Cytokine-Family Biomarker Candidates for Small Abdominal Aortic Aneurysm Identified via Integrated mRNA and Protein Expression Profiling

**DOI:** 10.3390/ijms27114863

**Published:** 2026-05-28

**Authors:** Piotr Stabiszewski, Daniel Zalewski, Przemysław Kołodziej, Marta Ziaja-Sołtys, Joanna Łuszczak, Magdalena Szymańska, Alicja Petniak, Jacek Bogucki, Piotr Terlecki, Barbara Stawarz, Janusz Kocki, Marcin Feldo, Anna Bogucka-Kocka

**Affiliations:** 1 Department of Vascular Surgery, St. Padre Pio Provincial Hospital in Przemyśl, 18 Monte Cassino St., 37-700 Przemyśl, Poland; stabisz@gmail.com; 2 Department of Biology and Genetics, Medical University of Lublin, 4a Chodźki St., 20-093 Lublin, Poland; 3 Clinical Dietetics Unit, Medical University of Lublin, 20-093 Lublin, Poland; 4 Department of Clinical Genetics, Medical University of Lublin, 11 Radziwiłłowska St., 20-080 Lublin, Poland; 5 Institute of Medical Sciences, The John Paul II (The Second) Catholic University of Lublin, Konstantynów 1F St., 20-708 Lublin, Poland; 6 Department of Vascular Surgery and Angiology, Medical University of Lublin, 8 Solidarności St., 20-841 Lublin, Poland; piotr.terlecki@umlub.edu.pl (P.T.);; 7 St. Padre Pio Provincial Hospital in Przemyśl, 18 Monte Cassino St., 37-700 Przemyśl, Poland

**Keywords:** abdominal aortic aneurysm, gene expression, plasma protein, biomarker

## Abstract

Abdominal aortic aneurysm (AAA) is a chronic vascular disease characterized by localized dilatation of the abdominal aorta. This condition is frequently underdiagnosed and carries a high mortality rate (65–85%) due to aneurysm rupture. Although numerous candidate biomarkers have been identified for AAA, none have been successfully implemented in clinical diagnostic procedures for AAA screening. This highlights the critical need for the discovery and validation of reliable biomarkers to improve early detection and risk stratification in AAA. Therefore, in our study, we aimed to identify small AAA (sAAA) biomarker candidates among the key cytokines and receptors of IL-1, IL-6, IL-8, IL-10, and IL-17 families on gene expression and plasma protein levels. Comparative analysis was conducted between a group of 100 men with sAAA (<54 mm in diameter) and a group of 100 men without AAA. Expression profiles of the analyzed cytokines and their receptors were obtained in peripheral blood mononuclear cells using real-time PCR, while plasma levels of selected encoded proteins were determined using ELISA. The mean expression of *IL10RA*, *IL17RA*, *CXCL8*, and *IL1B*, as well as the plasma levels of IL-17A, were significantly different between the sAAA and Control groups, with *CXCL8* and *IL1B* exhibiting a strong mutual correlation. The diagnostic model incorporating these biomarker candidates and D-dimer levels showed a fair classification performance (ROC-AUC = 0.756), with a sensitivity and specificity of approximately 0.7. The selected biomarker candidates were functionally associated with fibroblast activation, neutrophil chemotaxis, T-helper cell function, and cell adhesion and proliferation. Cytokines selected as biomarker candidates represent a promising field for further studies on the identification of diagnostic targets for the early detection of AAA.

## 1. Introduction

Abdominal aortic aneurysm (AAA) is defined as a localized pathological dilation of the abdominal aorta exceeding 50% of its normal diameter or reaching an absolute diameter of ≥3.0 cm. Most AAAs occur in the infrarenal segment (below the renal arteries), accounting for approximately 80% of all aortic aneurysms. The clinical severity, prognosis, and management strategies are primarily determined by the maximal aneurysm diameter. Aneurysms measuring 3.0–5.4 cm in diameter are generally classified as small AAAs (sAAAs), whereas large AAAs are those measuring ≥ 5.5 cm [1,2,3].

The global prevalence of AAA has been estimated at 0.92% among individuals aged 30–79 years [4], with sAAAs constituting most cases [5]. In screening studies conducted in Polish populations aged over 60 years, the prevalence of AAAs has been reported to range between 2.62% and 6%, with small aneurysms accounting for approximately 84–92% of all detected AAA cases [6,7,8].

Due to their largely asymptomatic course, AAAs remain substantially underdiagnosed and are frequently detected incidentally during imaging performed for unrelated indications. Progressive aneurysm enlargement may ultimately lead to rupture, which results in massive intra-abdominal hemorrhage, hemodynamic collapse, and is associated with mortality rates of up to 85%. Although the annual risk of rupture of sAAAs is estimated to be <1%, they exhibit an average growth rate between 2 and 3 mm/year, with rupture rates estimated to double for every 0.5 cm increase in diameter [1,2,3,9,10,11,12,13]. Moreover, patients with sAAA are burdened with a significant risk of developing cardiovascular events [14]. Current clinical guidelines recommend regular imaging surveillance for sAAAs using ultrasonography, as there is no evidence of an advantage to early repair interventions, regardless of the method used (open surgery or endovascular repair) [13,15,16,17,18,19].

Cytokines play a central role in AAA pathogenesis by orchestrating chronic vascular inflammation, extracellular matrix degradation, and apoptosis of vascular smooth muscle cells. Pro-inflammatory cytokines, such as interleukin (IL)-1β, IL-6, and IL-17, are dysregulated in aneurysmal tissue and circulation, contributing to the recruitment and activation of inflammatory cells. Chemokines, including IL-8 and other CXC/CC family members, further amplify this process by mediating leukocyte chemotaxis and sustaining local inflammatory responses. Although predominantly pro-inflammatory signaling drives AAA development, certain cytokines, such as IL-10, exhibit protective effects, highlighting the complex and context-dependent cytokine network involved in disease progression [20,21,22]. Together, dysregulated cytokine and chemokine signaling constitutes a key mechanistic pathway in AAA pathology and represents a promising field for the identification of potential diagnostic and therapeutic targets [23]. The recognition of inflammatory biomarkers reflecting the underlying pathophysiology may improve early diagnosis and risk prediction, supporting timely initiation of surveillance and risk factor optimization. Circulating biomarkers are particularly attractive owing to their feasibility and high potential for implementation in routine clinical practice.

Although numerous candidate inflammatory biomarkers for AAA have been investigated [20,24,25,26,27,28,29,30], none have achieved widespread clinical application because of their limited specificity, inconsistent reproducibility, or insufficient external validation. Most studies have focused on large AAAs, whereas only a few have identified biomarkers for sAAA. For example, multiple pro-inflammatory genes, including cytokines (*IL1B*, *IL6*, *IL8*), chemokines (*CXCL2*, *CXCL13*, *CCL3L3*, *CCL4L2*), lymphocyte activation antigens (*CD69*, *CD19*), and natural killer cell regulator *NKTR*, were found to be upregulated in aneurysmal aortic tissues of patients with sAAA [31]. Tumor necrosis factor-α (TNF-α) and POU class 2 homeobox associating factor 1 (POU2AF1) expression was upregulated in sAAA tissues, suggesting the involvement of B cell activation and macrophage infiltration in early stage AAA pathophysiology [32,33].

The locally ongoing inflammatory processes within the aneurysmal aortic wall may be at least partially reflected in the peripheral circulation, as several studies have identified circulating inflammatory markers associated with sAAA. For example, higher plasma levels of inflammation-related markers, including fibrinogen, high-sensitivity C-reactive protein (hsCRP), D-dimer, and dipeptidyl peptidase-4 activity, were found in the plasma of sAAA patients compared to non-AAA individuals [34,35,36]. In contrast, the mean plasma levels of catalase and its activity in polymorphonuclear neutrophils were lower in patients with sAAA [37]. Higher serum C-reactive protein (CRP) levels were associated with a significantly increased risk of major adverse cardiovascular events in patients with this disease [38]. There is still a critical need to identify novel biomarkers and rigorously validate previously proposed candidates.

**Figure 1:**
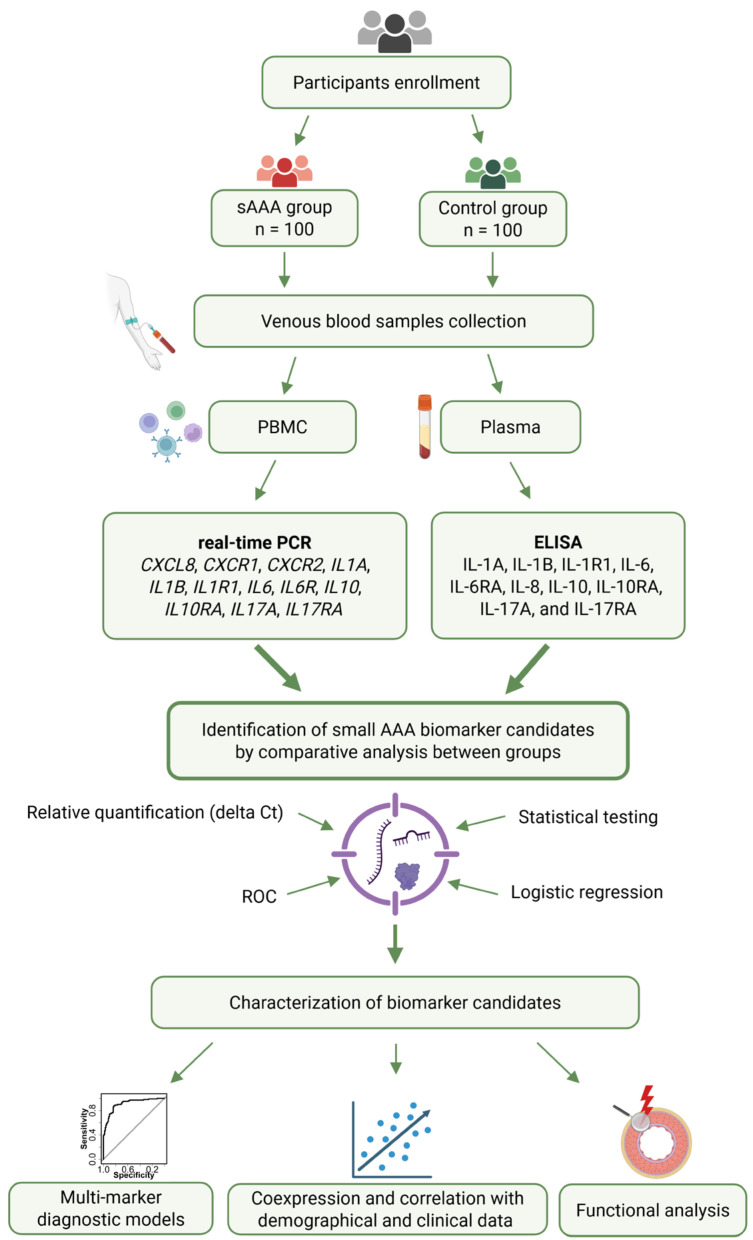
Outline of the study design. AAA—abdominal aortic aneurysm, sAAA—small abdominal aortic aneurysm, ELISA—enzyme-linked immunosorbent assay, PBMC—peripheral blood mononuclear cells, PCR—polymerase chain reaction, ROC—Receiver Operating Characteristic. Created in BioRender. Anna Bogucka-Kocka. (2026) https://BioRender.com/lghmk3u (accessed on 28 April 2026).

Consequently, in the present study, we performed an integrated analysis of peripheral blood mononuclear cell (PBMC) gene expression and encoded protein plasma levels of selected cytokines and their receptors to investigate their biomarker characteristics in a group of men with sAAA compared to non-AAA individuals. The study design is presented in Figure 1.

## 2. Results

### 2.1. Characterization of the Study Subjects

The study population included two groups of subjects: 100 patients with sAAA (sAAA group) and 100 volunteers without disease (Control group). Upon review of their clinical data, two individuals in the sAAA group were removed from further analysis due to aberrant blood laboratory tests results indicative of a potential underlying, previously unrecognized pathology. The demographic and clinical characteristics of the remaining participants are presented in Table 1. There were no significant differences between the sAAA and Control groups in terms of age, smoking, coronary artery disease (CAD), and C-reactive protein (CRP) and fibrinogen levels. Small, borderline differences were found in the BMI and the presence of lower extremity artery disease (LEAD). The sAAA group was characterized by significantly higher mean D-dimer levels. The mean AAA diameter in the sAAA group was 40.93 mm, and the most common location was the infrarenal aneurysm (87.8%).

### 2.2. Cytokine-Related Genes Are Differentially Expressed in PBMC of Individuals with sAAA

Expression profiles of 12 genes encoding cytokines and their receptors (*CXCL8*, *CXCR1*, *CXCR2*, *IL1A*, *IL1B*, *IL1R1*, *IL6*, *IL6R*, *IL10*, *IL10RA*, *IL17A*, and *IL17RA*) were determined in PBMC samples using real-time PCR and compared between 98 men with sAAA (≤54 mm, sAAA group) and 100 non-AAA men (Control group). During multi-step data quality control, 10 samples and 3 genes were excluded from the analysis due to low signal or unreliable data (see Section 4 for more details). The expression levels of the nine retained genes were compared between the sAAA and Control groups using the delta Ct method for relative quantification, Receiver Operating Characteristic (ROC) analysis, and univariate logistic regression. Genes characterized by fold change with FDR < 0.05, area under the ROC curve (ROC-AUC) > 0.6, and logistic regression odds ratio with FDR < 0.05 were considered as the most promising candidates for sAAA biomarkers. The size of the fold change was not employed as a criterion for biomarker candidate selection due to the early disease stage of the patient cohort, in which even subtle transcriptomic dysregulations may be biologically and clinically meaningful. Therefore, applying a fold-change threshold could result in the exclusion of potentially relevant biomarkers.

**Figure 2:**
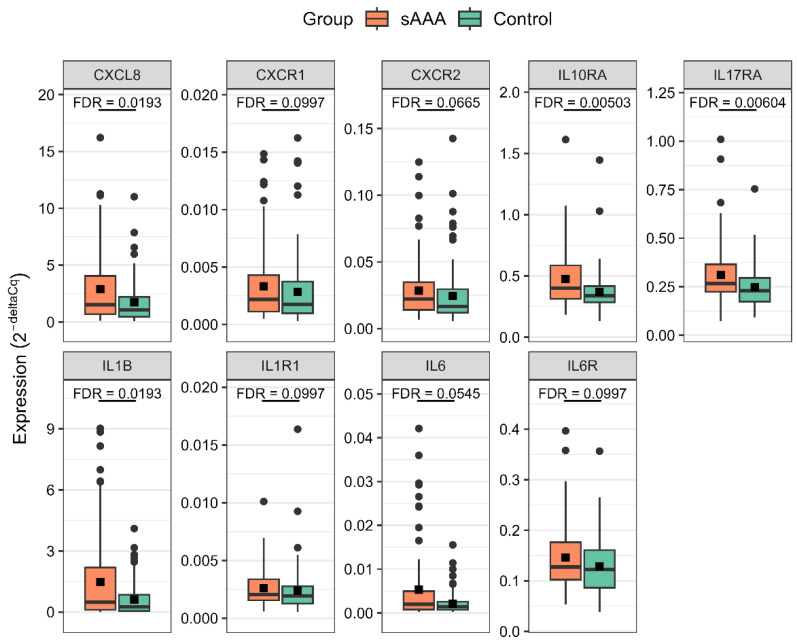
Distributions of normalized expression (2^−deltaCq^ values) of nine cytokine genes in the sAAA and Control groups. Whiskers reach the most distant point in the doubled interquartile range, and boxes range between the 25% and 75% quartiles. Horizontal lines and squares inside the boxes mark the median and mean values, respectively. FDR—false discovery rate (*p* values adjusted by Benjamini–Hochberg method), sAAA—small abdominal aortic aneurysm.

The application of established selection criteria allowed the selection of four biomarker candidate genes: *IL10RA*, *IL17RA*, *CXCL8*, and *IL1B* (Table 2). All selected genes exhibited statistically significantly higher mean expression levels in the sAAA group than in the Control group (Figure 2).

### 2.3. Mean Plasma Level of IL-17A in Individuals with sAAA Was Significantly Lower Compared to Non-AAA Controls

The ELISA method was used to analyze the plasma levels of 10 proteins (IL-1A, IL-1B, IL-1R1, IL-6, IL-6RA, IL-8, IL-10, IL-10RA, IL-17A, and IL-17RA) encoded by the genes analyzed in this study (see Section 2.2). Analysis was performed in plasma samples from 98 patients with sAAA (sAAA group) and 100 healthy controls (Control group), the same as that used for gene expression analysis. One protein with a high number of concentrations below the limits of detection, and two outlier samples were excluded from the analysis (see Section 4 for more details).

**Figure 3:**
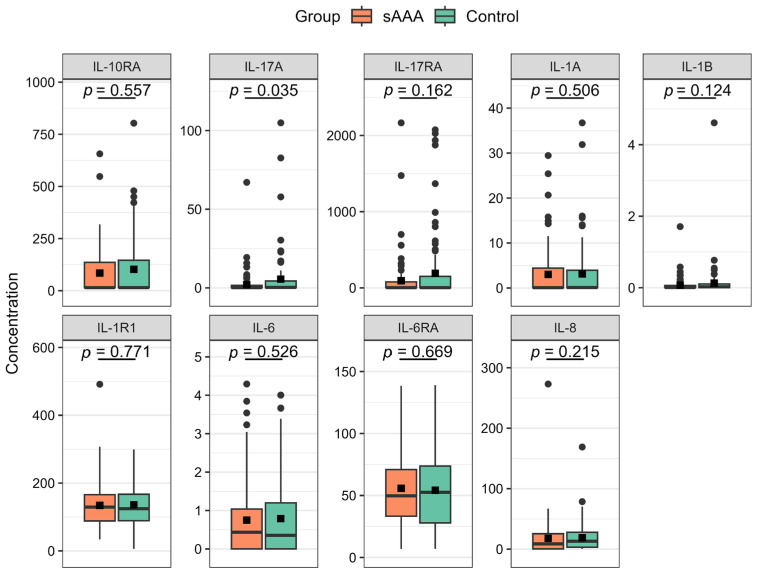
Distributions of plasma levels of nine cytokine proteins in patients with small abdominal aortic aneurysm (sAAA group) and non-AAA controls (Control group). Concentrations are presented in pg/mL, except for IL-1R1 and IL-6RA, which are shown in ng/mL. Whiskers reach the most distant point in the doubled interquartile range, while boxes range between the 25% and 75% quartiles. Horizontal lines and square inside boxes mark the median and mean values, respectively. *p*—statistical significance.

The distributions of the plasma levels of 9 remaining proteins are shown in Figure 3. The mean concentration of one protein, IL-17A, was significantly different (*p* = 0.035) between the sAAA and Control groups (Table 3). In the ROC analysis, the obtained ROC-AUC values indicated a low discriminative value, with IL-17A having the highest ROC-AUC of 0.58. The univariate odds ratios estimated for the analyzed cytokines did not reach statistical significance.

The results indicate that alterations in plasma IL-17A concentrations exhibit the strongest discriminative capacity among all analyzed proteins; therefore, IL-17A was the only protein considered as a potential biomarker candidate.

### 2.4. Multi-Marker Diagnostic Models Show Fair Classification Performance

**Figure 4:**
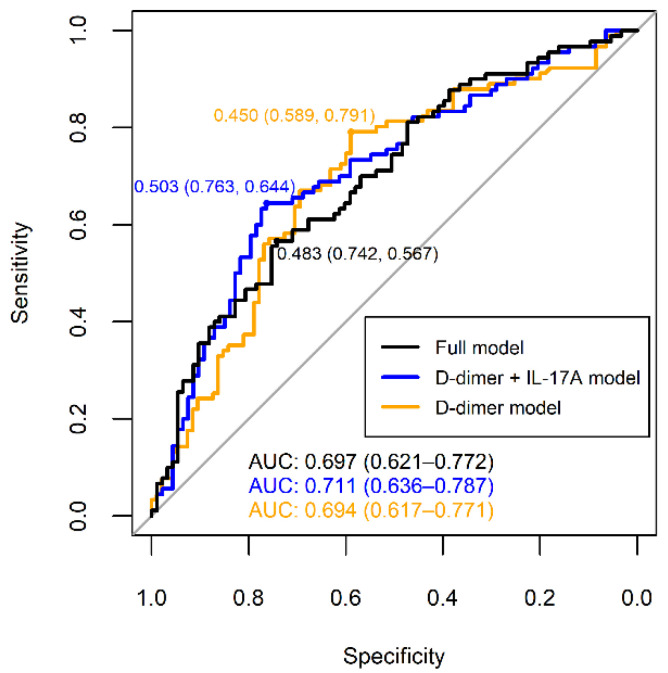
Diagnostic performance of small AAA patients classification by three models: full model with all predictors (D-dimer levels and expression of five biomarker candidates: *IL10RA*, *IL17RA*, *CXCL8*, and *IL1B* genes, and IL-17A protein, black color), model with D-dimer and IL-17A protein (blue color), and model with D-dimer alone (orange color). The plot presents AUC values together with 95% confidence intervals, as well as threshold values with corresponding specificity and sensitivity (in that order).

The combined diagnostic performance of the five selected biomarker candidates (*IL10RA*, *IL17RA*, *CXCL8*, *IL1B*, and IL-17A) was assessed using ROC analysis based on the probabilities generated by a multivariable logistic regression model. The D-dimer parameter was also included in the model, as its mean plasma levels differed significantly between the sAAA and Control groups (Table 1). The full model with all these variables resulted in ROC-AUC = 0.697 and accuracy = 0.656 at a threshold of 0.483 (Figure 4).

Furthermore, models incorporating different combinations of predictors were evaluated to identify an ‘economic’ model that included the minimal number of predictors while maintaining minimal loss of diagnostic performance. The final model included only the plasma levels of D-dimers and IL-17A protein. It exhibited a slightly higher ROC–AUC (0.711) and at a cut-off of 0.503 it achieved higher accuracy (0.705), as well as improved specificity and sensitivity, compared with the full model with all predictors (Figure 4).

Finally, a third model incorporating only the D-dimer parameter was constructed to compare its diagnostic performance with the two previously developed models. This D-dimer-only model demonstrated the lowest discriminative performance, with a ROC–AUC value of 0.694. However, the optimal cut-off threshold (0.450) was associated with the highest sensitivity (0.791), corresponding to the lowest specificity (0.589), compared with the multivariable models (Figure 4).

No statistically significant difference was observed in the ROC-AUC values between these models (*p* > 0.05, DeLong method [40]).

### 2.5. Co-Expression of Biomarker Candidates and Their Relationships with Demographical and Clinical Data

Pairwise correlation analysis was conducted among four selected cytokine genes (*IL10RA*, *IL17RA*, *CXCL8*, and *IL1B*) and the IL-17A protein, in order to evaluate potential associations between their expression levels. A strong positive correlation was found between the expression levels of *CXCL8* and *IL1B* (R = 0.94, *p* < 2.2 × 10^−16^), whereas a moderate positive relationship was found between *IL10RA* and *IL-17RA* (R = 0.63, *p* < 2.2 × 10^−16^) (Table S1, Figure S1). These correlations may indicate co-expression, functional association, and common regulatory mechanisms of correlated genes, potentially implicated in early AAA pathophysiology. Plasma IL-17A levels were not significantly correlated with PBMC expression of any of the analyzed genes (|R| < 0.15, Table S1).

Regarding correlations with demographic and clinical data, no significant correlations were observed for continuous-type variables (age, body mass index, and circulatory levels of C-reactive protein, fibrinogen, and D-dimers). All the obtained correlation coefficients (absolute R values) were <0.2 (Table S2). For categorical-type data (smoking and presence of CAD and LEAD), statistically significant associations were found only for *CXCL8* and *IL1B* genes (Table S3). These genes showed elevated expression in patients with atherosclerotic conditions (CAD and LEAD; Figure S2), suggesting that their potential involvement may represent a mechanistic link between atherosclerosis and aneurysm formation. This is consistent with one of the proposed hypotheses of aneurysm formation, which suggests that it represents a compensatory response to reduced vessel diameter and shear stress alterations resulting from the presence of atherosclerotic plaques [41,42].

### 2.6. Functional Analysis of Biomarker Candidates Revealed Strong Associations with Distinct Immune Response Signalling Pathways

**Figure 5:**
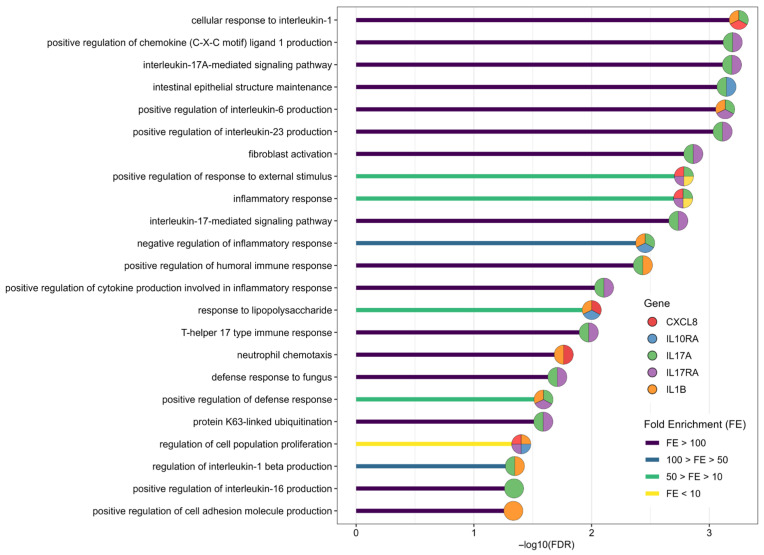
Biological processes associated with five candidate biomarkers of small abdominal aortic aneurysm: *IL10RA*, *IL17RA*, *CXCL8*, *IL1B*, and ^IL17A^ (encoding IL-17A protein).

The five selected biomarker candidates are members of the broad cytokine family, which plays a central role in initiating and regulating immune responses. To further explore their biological functions, a functional overrepresentation analysis was conducted using the Gene Ontology Biological Process database and PANTHER Classification System website tool [43] (Table S4). The lowest functional terms in the hierarchy with FDR < 0.05 are presented in Figure 5. Functional terms related to the positive regulation of the immune response involving biomarker candidates and other regulators, such as CXCL1, IL-6, IL-16, and IL-23, were disclosed. The obtained functional terms were associated with fibroblast activation, neutrophil chemotaxis, T-helper cell function, and cell adhesion and proliferation (Figure 5).

## 3. Discussion

The present study contributes to research efforts aimed at developing effective, noninvasive tools for the early diagnosis, prognosis, and risk stratification of AAA. The identification of circulating biomarkers that accurately reflect pathological processes within the aortic wall may complement and potentially enhance current gold-standard imaging modalities used in the diagnosis and monitoring of AAA.

We integrated gene and protein expression data to investigate key members of the IL-1, IL-6, IL-8, IL-10, and IL-17 cytokine families to identify potential circulating biomarkers of early stage AAAs (sAAAs). Specifically, we analyzed the PBMC expression profiles of 12 cytokines and their receptors (*CXCL8*, *CXCR1*, *CXCR2*, *IL1A*, *IL1B*, *IL1R1*, *IL6*, *IL6R*, *IL10*, *IL10RA*, *IL17A*, and *IL17RA*), and the plasma concentrations of 10 corresponding proteins (IL-1A, IL-1B, IL-1R1, IL-6, IL-6RA, IL-8, IL-10, IL-10RA, IL-17A, and IL-17RA) in cohorts comprising 98 sAAA patients and 100 non-AAA subjects. Candidate biomarkers were selected and characterized based on their co-expression patterns, associations with demographic and clinical parameters, and biological functions. In addition, their combined diagnostic utility was assessed by developing a multi-biomarker predictive models.

The current research literature contains only a limited number of studies focusing specifically on circulating biomarkers for human sAAAs. For example, metabolomic analysis performed using high-performance liquid chromatography-mass spectrometry (HPLC-MS) revealed numerous metabolites (e.g., sphinganine-1-phosphate) with high discriminatory potential between sAAA patients and controls [44]. Lower levels of tyrosine kinase receptor Axl and soluble tumor necrosis factor-like weak inducer of apoptosis (sTWEAK) [45,46], while higher levels of dipeptidyl peptidase-4 (CD26) and D-dimer have been previously found in the plasma of patients with sAAA [34,35,36]. Regarding studies focusing on inflammatory markers, lower polymorphonuclear neutrophil catalase activity and its plasma levels were found in patients with small and large AAA [37]. Furthermore, higher serum C-reactive protein (CRP) levels were associated with a significantly higher risk of major adverse cardiovascular events in individuals with sAAA [38]. Some hemostasis and inflammatory factors (e.g., tissue type plasminogen activator, IgA-antibodies against *Chlamydia pneumoniae*, alpha-1-antitrypsin, MMP9, and sTWEAK) have been associated with sAAA growth rate [45,47,48].

The current study contributes to these efforts by identifying cytokine family members that are differentially expressed in patients with sAAA and that may serve as potential diagnostic and prognostic targets. Inter-group comparative analysis revealed significantly higher expression of *IL10RA*, *IL17RA*, *CXCL8*, and *IL1B*, and lower plasma IL-17A concentrations in sAAA patients in relation to non-AAA control subjects (Table 2 and Table 3); therefore, these factors were identified as potential biomarker candidates for sAAAs.

Interestingly, *CXCL8* and *IL1B* exhibited a strong correlation, suggesting their functional association (Figure S2). Indeed, previous studies have demonstrated that these two factors act cooperatively during the inflammatory response. For instance, IL-1β promotes neutrophil recruitment by enhancing CXCL8 expression during the early inflammatory phase following *Streptococcus pneumoniae* infection [49]. In another study, neutralization of IL-1β released by murine peritoneal macrophages infected with *Staphylococcus aureus* suppressed CXCL8 and other cytokine secretion, ultimately impairing bacterial phagocytosis [50]. Furthermore, IL-1β and CXCL8 are key pro-inflammatory mediators with well-documented roles in aneurysm formation. Their activity is associated with enhanced infiltration of inflammatory cells, increased intracellular stress, and induction of neutrophil extracellular trap formation. Targeting these signaling pathways protects against aortic aneurysm development [51,52,53,54,55,56,57,58]. Finally, genes *IL1B* and *CXCL8* have been previously shown to be upregulated and positively correlated in the aortic wall of patients with AAA. Their functional interplay may involve the association of CXCL8 with NLRP3 inflammasome formation, which promotes caspase-1–dependent processing and secretion of IL-1β [51]. Therefore, it may be hypothesized that this mechanism may contribute to the maintenance of a sustained chemotactic and pro-inflammatory microenvironment within the aneurysmal aortic wall, thereby facilitating immune cell infiltration and activation of innate immune responses. This hypothesis can be further supported by the significant enrichment of these genes within the neutrophil chemotaxis functional term. Nevertheless, this proposed mechanism remains speculative and requires validation in further studies (Figure 5).

The localized upregulation of *IL1B* and *CXCL8* signaling in aneurysmal tissue is probably reflected in the increased expression of these genes in PBMC. Elevated *CXCL8* expression has been observed in PBMCs from patients with sAAAs (as demonstrated in the present study) and large AAA [54,59], suggesting that this alteration reflects both early and advanced stages of aneurysm development. In contrast, higher expression of *IL1B*, which has been consistently reported in AAA tissue samples [60], appears to be reflected in PBMCs primarily in patients with sAAA (as demonstrated in the present study), but not in those with large AAA [59]. These findings may suggest a stage-dependent pattern of systemic cytokine expression; however, further studies are warranted to assess the correlations in cytokine levels across different biological compartments to determine the extent to which localized pathological processes are mirrored in the peripheral circulation.

In addition, the expression levels of their corresponding receptors (*IL1R1*, *CXCR1*, and *CXCR2*) did not differ significantly between the sAAA and Control groups (Table 2), suggesting that the observed effects are primarily driven by ligand-dependent signaling rather than alterations in receptor expression.

The increased expression of *IL1B* and *CXCL8* in PBMCs observed in the present study may also be influenced by comorbid atherosclerotic conditions (Figure S2). This hypothesis is consistent with previous reports linking both *IL1B* and *CXCL8* to the pathogenesis of atherosclerosis [61,62].

Our study also implicates the pro-inflammatory IL-17 signaling pathway in the development of sAAA, as both PBMC-derived expression of *IL-17RA* and plasma levels of IL-17A were identified as biomarker candidates. Interestingly, these candidates exhibited opposing patterns of alteration, with increased expression of *IL-17RA* in PBMC (Table 2) and decreased levels of IL-17A (Table 3) in plasma in the sAAA group. This inverse relationship may suggest that the upregulation of cellular *IL-17RA* receptors facilitates the enhanced uptake of circulating IL-17A from the extracellular compartment. This hypothesis can be supported by a meta-analysis demonstrating significantly higher expression of IL-17 in aortic tissue, while circulatory levels are lower [60]. However, this hypothesis was not experimentally validated in the present study and should therefore be considered speculative pending further investigation. Furthermore, no significant differences in plasma IL-17 concentrations have been previously reported between small and large AAA cases [63], suggesting that circulating levels may not directly reflect disease progression.

Although previous studies have shown increased mRNA and protein expression of IL-17 in the aortic tissue of AAA patients [64,65,66], findings from animal models investigating the role of IL-17 in AAA remain inconsistent. For example, IL-17 knockout mice exhibited attenuated aneurysm development following elastase perfusion [64], whereas inhibition of IL-17 signaling exacerbated AAA severity in angiotensin II-infused mice [67,68]. These discrepancies suggest a complex and context-dependent role of IL-17 signaling in AAA pathogenesis. They may also reflect the methodological differences among experimental models, which only partially recapitulate the multifactorial nature of human AAA development [69].

As a pro-inflammatory cytokine, IL-17A has been implicated in the pathogenesis of infectious diseases, autoimmune disorders, and neoplastic conditions [70,71]. Elevated plasma levels of IL-17A have been reported in several inflammatory diseases, including periodontitis [72], atopic dermatitis [73], rheumatoid arthritis [74], and lung cancer [75]. In the context of vascular pathology, increased plasma IL-17 levels have been observed in acute thoracic aortic dissection [76], and IL-17 signaling has also been associated with aortic calcification [77] and atherosclerosis [78]. Against this background, the lower plasma levels of IL-17A observed in patients with AAA, although consistent with previous findings [79], appear unexpected given the inflammatory nature of AAA pathogenesis. The biological significance of this dysregulation remains unclear. In addition to the hypothesis concerning enhanced uptake of IL-17A mediated by increased expression of its receptors within aneurysmal lesions or circulating immune cells, the observed reduction may be influenced by additional factors, including pharmacological treatment or alterations in cytokine metabolism associated with coexisting comorbidities. However, these mechanisms remain speculative and require validation in further experimental and clinical studies.

The different direction of IL-17A dysregulation in AAA compared to that reported in several other inflammatory and vascular disorders, may represent a potential opportunity for differential discrimination of AAA from these diseases. Nevertheless, further studies are required to validate this hypothesis and to more comprehensively elucidate the role of dysregulated IL-17 signaling pathway in AAA pathophysiology.

Expression of *IL10RA* was found to be increased in PBMCs of patients with sAAA (Table 2), whereas plasma concentrations of its corresponding ligand, IL-10, were below the detection threshold of the ELISAs used. Previous studies have demonstrated that IL-10 exerts protective effects in experimental AAA models [79,80,81] and is downregulated in the serum and plasma of AAA patients, although no significant differences have been observed between small and large aneurysms [63,82]. Based on these findings, it may be hypothesized that the increased expression of *IL10RA* in PBMCs represents a compensatory response to reduced availability of its ligand IL-10. Additional investigations using more sensitive analytical approaches are required to validate this hypothesis in the future.

Upregulation of *IL10RA* has also been reported in vascular tissues from patients with atherosclerosis-associated intracranial aneurysms [83], suggesting that this molecule may represent a shared mechanistic link between the pathophysiology of intracranial and aortic aneurysms.

The concordance between the direction of expression changes observed for the biomarker candidates in circulating compartments and previously reported findings in aneurysmal tissue is summarized in Table 4, demonstrating consistent regulation for IL-1B, CXCL8, and IL-10RA, while for IL-17 and IL-17RA, further studies are required to clarify this relationship.

Investigating early stage AAA pathology and associated circulatory biomarkers is a significant challenge. The complex etiopathogenesis of AAA develops gradually within the aneurysmal wall and can, in principle, be effectively characterized using tissue-derived material. However, such material is not accessible at this stage, as surgical intervention is not recommended for sAAAs. Therefore, peripheral blood is an optimal research material that can be obtained at any point time. However, blood compartments do not fully reflect locally ongoing pathological changes in the aorta, and AAA-related molecular signatures are rather less distinguishable than in aortic tissue. Accordingly, in the current study, the biomarker candidates identified in PBMC and plasma exhibited fair individual discriminative performance, with the highest diagnostic accuracy observed for *IL10RA* gene expression (ROC–AUC = 0.65, Table 2). Presented molecular dysregulations, although subtle, may reflect a systemic signature of aortic pathology, indicating the mechanisms involved in the early stages of aneurysm development.

To enhance the diagnostic utility of the selected biomarker candidates, we developed multivariable models integrating complementary biomarkers, aiming to improve performance beyond that achievable with single-marker approaches. The initial full model incorporated all five candidate biomarkers together with D-dimers, which demonstrated a significant difference between the sAAA and Control groups (Table 1). The inclusion of D-dimers in the diagnostic model is further supported by their established association with both the presence and progression of AAA-related diseases, including subaneurysmal aortic dilation and sAAA. Moreover, previous studies have highlighted its strong discriminatory capacity in distinguishing AAA from other atherosclerotic diseases, including coronary artery disease and peripheral artery disease, underscoring the relevance of D-dimer indicator as both a diagnostic and prognostic biomarker [36,84,85,86,87,88,89,90].

This full multivariable model demonstrated fair diagnostic performance, achieving a receiver operating characteristic area under the curve (ROC–AUC) of 0.697. At the selected optimal cut-off threshold, the model exhibited relatively high specificity (>0.74) but comparatively low sensitivity (0.57), suggesting a greater ability to correctly identify non-AAA individuals than to detect all affected cases (Figure 4). However, the inclusion of six variables limits the model’s feasibility and clinical applicability. Therefore, reducing the number of parameters to a minimal set of the most informative markers is desirable to enhance practicality while preserving diagnostic accuracy. Accordingly, a simplified, more “economical” model was constructed, incorporating only D-dimer and IL-17A plasma levels. This model maintained an improved diagnostic performance with slightly higher sensitivity and specificity at the selected decision threshold (Figure 4). The third model, incorporating only the D-dimer parameter, demonstrated the lowest ROC–AUC value among the evaluated models, while the selected optimal cut-off threshold yielded the highest sensitivity (0.79). However, the differences in diagnostic performance between the three models did not reach statistical significance.

Overall, although the inclusion of the selected biomarker candidates alongside D-dimer resulted in improvement in diagnostic performance, the difference was relatively small. Although D-dimer retains potential utility as a sensitive screening marker for sAAA, it is a highly nonspecific biomarker, as elevated levels can occur in a wide range of physiological and pathological conditions, including pregnancy, malignancies, and infections [91]. Therefore, the incorporation of additional, potentially more disease-specific biomarkers may enhance the overall diagnostic accuracy and specificity for sAAA. Further large-scale clinical studies involving well-characterized patient cohorts are required to optimize the selection of predictors for the detection of sAAA and to validate their potential clinical applicability.

In the present study, three genes (*IL17A*, *IL1A*, and *IL10*) were excluded from analysis due to insufficient data quality. Specifically, Ct values were either undetectable by the real-time PCR system or were derived from amplification curves of inadequate quality, precluding reliable quantification.

Interestingly, although the expression of *IL17A* and *IL1A* transcripts could not be consistently quantified in PBMC samples, the corresponding proteins were successfully detected in the majority of plasma samples. In contrast, neither gene expression nor circulating protein levels of IL-10 could be reliably determined in PBMCs and plasma, respectively. One potential explanation for these findings is the intrinsically low basal expression of these cytokines in native, non-stimulated PBMCs, resulting in transcript levels below the reliable detection threshold of the applied real-time PCR methodology. This interpretation can be supported by previous studies demonstrating very low constitutive expression of *IL17A* and *IL10* in unstimulated PBMCs and their cellular subsets [92,93,94,95,96].

At the same time, insufficient analytical sensitivity of the applied methodologies cannot be excluded as a contributing factor limiting detection capability. This possibility may be particularly relevant for the protein-level analyses of IL-17A and IL-10, whose circulating concentrations have been successfully quantified in previous studies [79,97]. Therefore, the weak or undetectable signals observed in the present study may, at least partially, reflect limited sensitivity of the ELISAs employed. Future investigations utilizing more sensitive transcriptomic and proteomic approaches may help clarify whether these cytokines are expressed at extremely low levels in circulation or whether their apparent absence primarily reflects technical limitations of the applied detection methods.

This study has several limitations. First, the sample size of the sAAA group was determined to be representative only for the population of Polish men. Consequently, further studies are required to validate the proposed biomarkers in women and diverse ethnic and geographic populations to determine whether their potential applicability is universal or population-specific. The absence of an external validation cohort confers an exploratory character to the present study and limits the generalizability of the findings. Consequently, the diagnostic utility of the proposed biomarker candidates should be interpreted with caution and requires validation in independent studies involving larger patient cohorts. Furthermore, the analysis was restricted to major representatives of the IL-1, IL-6, IL-8, IL-10, and IL-17 cytokine families, without assessing upstream regulators or downstream effector molecules. Comprehensive profiling of these pathways is necessary in future studies to achieve a deeper understanding of the molecular mechanisms underlying aneurysm development. Finally, the interpretations and mechanistic hypotheses proposed in Section 3 should be considered exploratory and require confirmation in future experimental and clinical investigations.

## 4. Materials and Methods

### 4.1. Outline of the Study Design

This study aimed to identify potential biomarkers of sAAA among selected cytokines (*CXCL8*, *IL1A*, *IL1B*, *IL6*, *IL10*, and *IL17A*) and their receptors (*CXCR1*, *CXCR2*, *IL1R1*, *IL6R*, *IL10RA*, and *IL17RA*) on gene expression and encoded protein levels. Two groups of subjects (100 men with sAAA and 100 non-AAA controls) were established for this study. Cytokine gene expression profiles were determined in PBMC using real-time PCR, while proteins were quantified in plasma using ELISA. The obtained results were compared between groups of participants, and the most promising biomarker candidates were selected by assessing their diagnostic potential using statistical testing of inter-group differences, ROC analysis, and logistic regression. Multi-marker diagnostic models were constructed using logistic regression predictions. Selected biomarker candidates were subjected to further analysis to explore their coexpression and correlations with demographic and clinical data. Functional analysis was also performed to estimate the role of selected biomarker candidates in AAA development. The outline of the study design is shown in Figure 1.

This study was conducted in accordance with the Declaration of Helsinki and approved by the Bioethics Committee of the Medical University of Lublin (decision no. KB-0024/111/07/2025). Informed and signed consent was obtained from all study subjects.

### 4.2. Study Participants

Two groups of men aged ≥ 45 years were enrolled in the study: 100 men with sAAA (sAAA group) and 100 men without AAA (Control group). The participants’ qualifications were carried out by experienced vascular surgeons (M.F. and P.S.) in two clinical hospitals in East Poland: University Clinical Hospital No. 1 in Lublin and St. Padre Pio Provincial Hospital in Przemyśl.

In the sample size calculations, when assumed that approximately 3% of the adult population in Poland present with sAAA [6,7,8], a group size of 100 individuals yields a low margin of error below 5% (4.4%) at a confidence level of 99%. The statistical power of two-tailed Student’s *t*-test for comparing two independent groups (100 participants each) was high as 0.9999 at assumptions of an effect size of 0.8 and an alpha level of 0.05.

Men who agreed to participate in the study underwent abdominal aorta examination by ultrasonography. Individuals diagnosed with sAAA with a maximum diameter of 54 mm were enrolled in the sAAA group, while men without abdominal aortic abnormalities were assigned to the Control group. The exclusion criteria included the presence of an inflammatory aneurysm, false aneurysm, thoracic aortic aneurysm, isolated popliteal or iliac artery aneurysm, and aortic and/or arterial dissection. As PBMC were used as the study material, individuals with conditions known to significantly affect immune cell function were excluded. These included connective tissue disorders (e.g., rheumatologic diseases), ongoing corticosteroid therapy, infection within the preceding six weeks, and hematological malignancies.

Venous blood samples (7.5 mL) were collected from all qualified participants using S-Monovette EDTA K3E blood collection tubes (Sarstedt, Nümbrecht, Germany). Demographic and medical data, along with laboratory test results (CRP, fibrinogen, and D-dimer blood levels), were collected from all the enrolled participants (Table 1). Laboratory tests were performed by the participating hospitals’ laboratory diagnostic departments using additional blood samples collected during the qualification procedure for the study. After data review, two patients in the sAAA group were excluded from the analysis due to abnormal results of laboratory blood tests, indicating potential diseases that had not been previously diagnosed.

### 4.3. Plasma Separation, PBMC Isolation and RNA Extraction

Immediately after blood sample collection, plasma was separated by centrifugation (2000× *g* for 10 min at room temperature), aliquoted, and stored at −80 °C for ELISA experiments. The remaining blood was used for PBMC isolation using gradient centrifugation with Gradisol L reagent (Aqua-Med, Łódź, Poland) according to the standard procedure (previously described in [98]). PBMC samples were subjected to total RNA extraction using the mirVana miRNA Isolation Kit (Invitrogen, Carlsbad, CA, USA), according to the manufacturer’s procedure. The quality and quantity of total RNA samples were assessed using a NanoDrop ND-1000 spectrophotometer (ThermoFisher Scientific, Waltham, MA, USA), Qubit 2.0 Fluorometer with the Qubit RNA High Sensitivity Assay Kit (both ThermoFisher Scientific, Waltham, MA, USA), and 4150 TapeStation instrument with RNA Screen Tape kit (both Agilent Technologies, Santa Clara, CA, USA). Total RNA samples with a 260/280 ratio higher than 1.8, 260/230 ratio higher than 1.5, and RNA integrity number (eRIN) greater than 7, were approved for further experiments.

### 4.4. Real-Time PCR for Gene Expression Analysis

Expression levels of 12 cytokine-related genes (*CXCL8*, *CXCR1*, *CXCR2*, *IL1A*, *IL1B*, *IL1R1*, *IL6*, *IL6R*, *IL10*, *IL10RA*, *IL17A*, and *IL17RA*) were quantified in the PBMC of subjects of sAAA and Control groups using real-time PCR.

Total RNA samples were reverse transcribed to obtain cDNA using the High Capacity cDNA Reverse Transcription Kit (Applied Biosystems, Foster City, CA, USA) according to the manufacturer’s protocol. PCR reactions were prepared in 384-well Custom TaqMan Gene Expression Array Cards using TaqMan Fast Advanced Master Mix and TaqMan Gene Expression Assays specific to target genes (all Applied Biosystems, Foster City, CA, USA), according to the manufacturer’s procedure. The assays used are listed in Table 5. *B2M*, *GAPDH*, and *YWHAZ* genes were also included to the analysis as a potential endogenous controls.

The PCR reactions were carried out in triplicates using the QuantStudio 7 Pro Real-Time PCR System (Applied Biosystems, Foster City, CA, USA) and following amplification protocol: uracil-N-glycosylase incubation (50 °C for 2 min), enzyme activation (92 °C for 10 min), and 40 amplification cycles (95 °C for 1 s and 60 °C for 20 s in each cycle).

Raw expression data were initially analyzed using QuantStudio Design & Analysis Software 2.8.0 (ThermoFisher Scientific, Waltham, MA, USA). Expression levels of the analyzed genes were determined as Cq values. Cq data along with quality control parameters, including AMPSCORE, Cq Confidence, and Amp.Status parameters, were subsequently imported to R 4.5.2 programming software [98]. Further analysis was performed using the RQdeltaCT 1.3.3 package and its dependent packages [99].

To retain only reliable Cq data determined from high-quality-shaped amplification curves, the Cq values higher than 33, with the parameter AMPSCORE < 1, Cq Confidence < 0.8, and with Amp.Status flag “Inconclusive” or “No Amp,” were filtered out. Moreover, Cq values flagged as “Undetermined” were excluded from the analysis. The amount of filtered data was similarly distributed across the samples (Figure S3); therefore, no samples were removed in this step. Regarding filtering across genes, those with filtered data in more than half of the subjects in at least one of the study groups were excluded from the study. These genes were *IL17A*, *IL1A*, and *IL10* (Figure S4). Subsequently, the data were subjected to collapsing of technical replicates and imputation of missing values (2.53% of the data) by mean values.

Differential expression analysis between the sAAA and Control groups was performed using the delta Ct method for relative quantification [100,101]. Briefly, the Cq values of the target genes were normalized using the Cq values of *GAPDH*, which showed the highest stability compared to the other housekeeping genes tested (*B2M* and *YWHAZ*) (Figure S5, Table S5). Subsequently, to improve the interpretability of the data, normalized Cq data (delta Cq values) were transformed to have a linear, monotonic scale using the 2^−deltaCq^ formula.

The uniformity of the transformed data was assessed using principal component analysis (PCA), which showed the outlier characteristics of the 10 samples. These samples were excluded from the analysis, which substantially improved data homogeneity (Figure S6).

The differences in the expression of the analyzed genes were expressed as fold-change values calculated by dividing the mean of the 2^−deltaCq^ values in the sAAA group by the mean of the 2^−deltaCq^ values in the Control group. The statistical analysis and further evaluation of the diagnostic values are described in Section 4.6.

### 4.5. ELISA Experiments

The concentrations of 10 proteins (IL-1A, IL-1B, IL-1R1, IL-6, IL-6RA, IL-8, IL-10, IL-10RA, IL-17A, and IL-17RA) encoded by the analyzed genes (refer to the chapter above) were determined in plasma collected from individuals in the sAAA and Control groups, the same as for the gene expression analysis. Plasma protein quantification was carried out using enzyme-linked immunosorbent assay (ELISA) kits purchased from Invitrogen (Carlsbad, CA, USA) and Abbexa (Cambridge, UK), according to the manufacturers’ instructions (Table 6).

ELISA measurements were performed in a blinded manner with respect to the case/control status of the samples. Plasma aliquots were thawed and centrifuged at 2000× *g* for 10 min at 4 °C and, if necessary, were diluted using the diluent buffer provided with the ELISA kit. Eight standard concentrations (for the standard curve) and blank wells containing a dilution buffer were used for each ELISA experiment. The prepared ELISA plates were read at the appropriate wavelength using a Synergy H1 microplate reader (BioTek, Winooski, VT, USA).

Data analysis was performed using Gen5 version 3.10 software (BioTek, Winooski, VT, USA) and R 4.5.2 programming software [100]. The background absorbance measured in the blank wells was subtracted from the readings of all other wells. Protein concentrations were then determined by interpolation from standard calibration curves. For diluted samples, the calculated values were adjusted by the appropriate dilution factor to obtain the final concentrations. The numbers of samples with concentration determined or not (above or below the limit of detection) are shown in Figure S7. If the protein concentration fell below the limit of detection, the values were set to the value of this limit. IL-10 exhibited an exceptionally high number of not determined values (Figure S7); therefore, this protein was excluded from the analysis. The homogeneity of the data was assessed using PCA (Figure S8), which detected two outlier samples that were removed from further analysis. Differences in plasma protein levels between the sAAA and Control groups were evaluated using statistical testing (see Section 4.6).

### 4.6. Statistical Analysis

The statistical analysis was performed using the R 4.5.2 programming environment. Statistical tests and methods were chosen depending on the type and distribution of the analyzed variables.

For continuous-type variables, the means were compared between the appropriate sample groups using a statistical test chosen based on data normality, which was assessed using the Shapiro–Wilk test (shapiro.test function in R). If the distributions of the data in both compared groups were defined as normal (*p* ≥ 0.05 in the Shapiro–Wilk test), the two-sided parametric Student’s *t*-test was used (*t*.test function in R). If the distributions of the expression values in at least one of the compared groups were defined as not normal (*p* < 0.05 in the Shapiro–Wilk test), the two-sided non-parametric Mann–Whitney U test (wilcox_rest function in coin 1.4-3 package) was used.

For categorical-type variables (contingency tables), the two-sided Chi-square test was used for inter-group comparisons (chisq_test function in coin 1.4-3 package).

Univariate and multivariate logistic regression was performed using the glm function in R. ROC analysis was performed using the pROC 1.18.5 [102] and caret 7.0-1 [103] packages. Ten-fold cross-validation was performed to reduce overfitting.

Relationships between continuous variables were investigated using correlation and linear regression methods. Correlation analysis was performed using the Spearman rank correlation test implemented in the Hmisc 5.1-2 package (https://cran.r-project.org/web/packages/Hmisc/index.html, accessed on 20 May 2026). Univariate linear regression models were constructed using the lm function in R.

Where indicated, *p* values were corrected for multiple testing using the Benjamini–Hochberg method to obtain FDR values. Results obtained with *p* or FDR < 0.05 were considered statistically significant. Visualizations were generated using the ggplot2 3.4.2 package (https://ggplot2.tidyverse.org/, accessed on 20 May 2026) in R, unless otherwise indicated.

Functional analysis was performed through Gene Ontology Biological Process terms using the Panther Classification System 19.0 website tool [43]. Functional terms in the lowest position in the hierarchy (the most specific) obtained from a statistical overrepresentation test (Fisher’s exact test) with FDR < 0.05 were retrieved. All genes from the whole human genome were used as a reference.

## 5. Conclusions

The comparative analysis performed between the sAAA and control groups for key representative cytokines of the IL-1, IL-6, IL-8, IL-10, and IL-17 families revealed differential PBMC expression of *IL10RA*, *IL17RA*, *CXCL8*, and *IL1B*, as well as altered plasma levels of IL-17A, identifying these molecules as potential biomarker candidates for early-stage AAA. The diagnostic model constructed using plasma IL-17A levels and D-dimer concentrations demonstrated optimal but fair discriminative performance, likely reflecting the inclusion of patients with sAAAs, in whom only initial pathophysiological alterations are present.

The selected biomarker candidates exhibited functional relevance to AAA pathogenesis and were associated with fibroblast activation, neutrophil chemotaxis, T-helper cell activity, and cell adhesion and proliferation. Moreover, the observed alterations in the PBMC expression of the three candidates, *IL1B*, *CXCL8*, and *IL10RA*, appear to be consistent with regulatory patterns previously reported in aneurysmal tissue, supporting their potential pathophysiological significance.

Further investigations are required to validate the diagnostic utility of the proposed candidates and verify the hypotheses proposed in Section 3. Signaling pathways associated with these candidates represent a promising direction for future efforts aimed at identifying biomarkers suitable for early-stage AAA detection.

## 6. Patents

A part of the results presented in this article has been included in patent applications No. P.455540 and P.455543 submitted to the Patent Office of the Republic of Poland.

## Figures and Tables

**Table 1:** Demographic and clinical data of the study participants.

Characteristic	sAAA Group (*n* = 98)	Control Group (*n* = 100)	*p* Value ^1^
Age	71.2 ± 6.35 (55–86)	69.2 ± 9.28 (45–94)	>0.05
Body mass index (BMI)	28.4 ± 3.89 (17.5–37.1)	27.5 ± 4.23 (17.5–41.5)	4.37 × 10^−2^
Smoking	33 (33.7%)	27 (27%)	>0.05
Coronary Artery Disease	17 (17.3%)	10 (10%)	>0.05
Lower Extremity Artery Disease	26 (26.5%)	42 (42%)	2.19 × 10^−2^
C-reactive protein (mg/L)	2.055 [0.885–4.535] (0.6–212)	1.820 [1.000–3.275] (0.6–51.4)	>0.05
Fibrynogen (g/dL)	373.7 ± 100.9 (147–716)	356.9 ± 97.2 (200–660)	>0.05
D-dimers (mg/L FEU)	1091 [694–1624] (215–32,713)	554 [342–979] (215–12,773)	1.31 × 10^−7^
AAA diameter (mm)	40.93 ± 5.90 (30–54)	Not applicable	
AAA localisation:			
Infrarenal	86 (87.8%)		
Infrarenal-iliac	4 (4.1%)		
Thoracoabdominal	4 (4.1%)		
Pararenal	2 (2%)		
Juxtarenal	1 (1%)		
Suprarenal	1 (1%)		

Continuous-type variables are presented as median [interquartile range] (for CRP and D-dimers) or mean ± SD (for remaining variables), as well as range in parenthesis. Categorical-type variables are presented as counts and percentages in brackets. ^1^ Statistical significance of differences between the group of patients with small abdominal aortic aneurysm (sAAA group) and the group of control subjects (Control group). The statistical tests used are described in Section 4.6 of this paper. FEU—fibrinogen equivalent unit, sAAA—small abdominal aortic aneurysm.

**Table 2:** Differential expression data of nine cytokine genes between small AAA and Control groups.

Gene Symbol	Gene Name	Relative Quantification		ROC	Univariate Logistic Regression	
		FCh	FDR	ROC-AUC	OR	FDR
*IL10RA* ^1^	interleukin 10 receptor subunit alpha	1.28	5.03 × 10^−3^	0.65	3.47	6.61 × 10^−3^
*IL17RA* ^1^	interleukin 17 receptor A	1.26	6.04 × 10^−3^	0.64	3.10	7.32 × 10^−3^
*CXCL8* ^1^	C-X-C motif chemokine ligand 8	1.66	1.93 × 10^−2^	0.61	1.54	7.79 × 10^−3^
*IL1B* ^1^	interleukin 1 beta	2.39	1.93 × 10^−2^	0.61	1.53	6.61 × 10^−3^
*IL6*	interleukin 6	2.49	5.45 × 10^−2^	0.59	1.62	7.79 × 10^−3^
*CXCR2*	C-X-C motif chemokine receptor 2	1.16	6.65 × 10^−2^	0.58	1.24	2.97 × 10^−1^
*CXCR1*	C-X-C motif chemokine receptor 1	1.18	9.97 × 10^−2^	0.57	1.17	3.15 × 10^−1^
*IL1R1*	interleukin 1 receptor type 1	1.08	9.97 × 10^−2^	0.57	1.17	4.53 × 10^−1^
*IL6R*	interleukin 6 receptor	1.14	9.97 × 10^−2^	0.57	1.97	7.79 × 10^−2^

^1^—genes selected as candidates for small AAA biomarkers. The provided gene symbols and names are in accordance with the nomenclature of the HUGO Gene Nomenclature Committee (HGNC) (https://www.genenames.org/, accessed 2 March 2026). AAA—abdominal aortic aneurysm, FCh—fold change, FDR—false discovery rate (*p* values adjusted by Benjamini–Hochberg method), OR—odds ratio, ROC—receiver operating characteristics, ROC-AUC—area under receiver operating characteristics curve.

**Table 3:** Differences in analyzed plasma protein levels between the small AAA and Control groups.

Protein Symbol	Protein Name	Mean Concentration ^1^		*p*	ROC-AUC
		sAAA	Control		
IL-17A ^2^	Interleukin-17A	2.10 ± 7.39	5.54 ± 15.24	0.035	0.58
IL-1B	Interleukin-1 beta	0.07 ± 0.19	0.13 ± 0.48	0.124	0.56
IL-17RA	Interleukin-17 receptor A	93.85 ± 281.78	191.11 ± 435.65	0.162	0.45
IL-8	Interleukin-8	17.90 ± 31.39	18.82 ± 22.66	0.215	0.55
IL-1A	Interleukin-1 alpha	3.01 ± 5.42	3.13 ± 5.86	0.506	0.53
IL-6	Interleukin-6	0.75 ± 0.94	0.79 ± 1.07	0.526	0.53
IL-10RA	Interleukin-10 receptor subunit alpha	84.75 ± 110.53	102.39 ± 140.95	0.557	0.48
IL-6RA	Interleukin-6 receptor subunit alpha	55.76 ± 29.20	54.21 ± 30.60	0.669	0.48
IL-1R1	Interleukin-1 receptor type 1	134.27 ± 67.03	135.80 ± 61.27	0.771	0.49

The provided protein names are in accordance with the UniProt database (release 2026_01) [39]. ^1^ pg/mL, except for IL-1R1 and IL-6RA, which are shown in ng/mL units, ^2^—protein selected as candidate biomarker of small AAA, *p*—statistical significance calculated by two-sided U Mann–Whitney test, ROC-AUC—area under receiver operating characteristics curve.

**Table 4:** Comparison of alterations in the expression of cytokines proposed as small AAA biomarker candidates in PBMCs, plasma, and aneurysmal tissue.

Tissue	Cytokines Proposed as Biomarker Candidates				
	IL-1B	CXCL8	IL-10RA	IL-17	IL-17RA
PBMC expression (our study)	↑	↑	↑	nd.	↑
Plasma concentration (our study)	ns.	ns.	ns.	↓	ns.
Aneurysm tissue (previous studies)	↑ [51]	↑ [51]	↑ [83]	↑ [60]	nd.

↑—higher expression, ↓—lower expression, ns.—determined but not statistically significant, nd.—not determined.

**Table 5:** TaqMan Gene Expression Assays used for the study.

Assay ID	Gene Symbol	Gene Name	Chromosome
Hs00187842_m1	*B2M*	beta-2-microglobulin	15
Hs00174103_m1	*CXCL8* (previously *IL8*)	C-X-C motif chemokine ligand 8	4
Hs00174146_m1	*CXCR1* (previously *IL8RA*)	C-X-C motif chemokine receptor 1	2
Hs00174304_m1	*CXCR2* (previously *IL8RB*)	C-X-C motif chemokine receptor 2	2
Hs99999905_m1	*GAPDH*	glyceraldehyde-3-phosphate dehydrogenase	12
Hs00961622_m1	*IL10*	interleukin 10	1
Hs00155485_m1	*IL10RA*	interleukin 10 receptor subunit alpha	11
Hs00174383_m1	*IL17A*	interleukin 17A	6
Hs01056316_m1	*IL17RA*	interleukin 17 receptor A	22
Hs00174092_m1	*IL1A*	interleukin 1 alpha	2
Hs01555410_m1	*IL1B*	interleukin 1 beta	2
Hs00991010_m1	*IL1R1*	interleukin 1 receptor type 1	2
Hs00174131_m1	*IL6*	interleukin 6	7
Hs01075664_m1	*IL6R*	interleukin 6 receptor	1
Hs00237047_m1	*YWHAZ*	tyrosine 3-monooxygenase/tryptophan 5-monooxygenase activation protein zeta	8

Provided gene symbols and gene names are in accordance with actual nomenclature in HUGO Gene Nomenclature Committee (HGNC) (https://www.genenames.org/, accessed 2 March 2026).

**Table 6:** ELISA kits used in the study.

Protein Symbol	Protein Name	Catalog Number	Quantitative Range	Analytical Sensitivity
IL-1A	Interleukin-1 alpha	BMS243-2	1.6–100 pg/mL	1.1 pg/mL
IL-1B	Interleukin-1 beta	BMS224-2HS	0.16–10 pg/mL	0.05 pg/mL
IL-1R1	Interleukin-1 receptor type 1	EHIL1R1	6–2000 pg/mL	6 pg/mL
IL-6	Interleukin-6	BMS213-2	1.56–100 pg/mL	0.92 pg/mL
IL-6RA	Interleukin-6 receptor subunit alpha	BMS214INST	78–5000 pg/mL	0.01 ng/mL
IL-8	Interleukin-8	KAC1301	7–750 pg/mL	0.7 pg/mL
IL-10	Interleukin-10	BMS215-2HS	0.39–25.0 pg/mL	0.03 pg/mL
IL-10RA	Interleukin-10 receptor subunit alpha	abx151923	2.05–500 ng/mL	2.5 ng/mL
IL-17A	Interleukin-17A	BMS2017	1.6–100 pg/mL	0.5 pg/mL
IL-17RA	Interleukin-17 receptor A	EHIL17R	3.2–3000 pg/mL	3.2 pg/mL

Provided protein names are in accordance with the actual nomenclature in the UniProt database (release 2026_01) [39].

## Data Availability

The raw data supporting the conclusions of this article will be made available by the authors on request.

## Supplementary Materials

The following supporting information can be downloaded at: https://www.mdpi.com/article/10.3390/ijms27114863/s1.
