# Supplementary material for: Cytokine-Family Biomarker Candidates for Small Abdominal Aortic Aneurysm Identified via Integrated mRNA and Protein Expression Profiling
Source: Int J Mol Sci. 2026 May 28;27(11):4863. doi: 10.3390/ijms27114863 (PMC13256898; doi:10.3390/ijms27114863)
Supplement: Supplementary file 1 [file ijms-27-04863-s001.zip › Supplementary Figures.pdf]

SUPPLEMENTARY FIGURES

Cytokine-Family Biomarker Candidates for Small Abdominal Aortic Aneurysm Identified Via Integrated mRNA and Protein Expression Profiling

Piotr Stabiszewski [1], Daniel Zalewski [2,*], Przemysław Kołodziej [2], Marta Ziaja-Sołtys [2], Joanna Łuszczak [2], Magdalena Szymańska [4], Alicja Petniak [5], Jacek Bogucki [6], Piotr Terlecki [3], Barbara Stawarz [7], Janusz Kocki [5] , Marcin Feldo [3], and Anna Bogucka-Kocka [2,*]

[1] Department of Vascular Surgery, St. Padre Pio Provincial Hospital in Przemyśl, 18 Monte Cassino St., 37-700 Przemyśl, Poland
[2] Chair and Department of Biology and Genetics, Medical University of Lublin, 4a Chodźki St., 20-093 Lublin, Poland
[3] Clinical Dietetics Unit, Medical University of Lublin, 20-093 Lublin, Poland
[4] Department of Clinical Genetics, Chair of Medical Genetics, Medical University of Lublin, 11 Radziwiłłowska St., 20-080 Lublin, Poland
[5] Institute of Medical Sciences, The John Paul II (The Second) Catholic University of Lublin, Konstantynów 1F St., 20-708 Lublin, Poland
[6] Chair and Department of Vascular Surgery and Angiology, Medical University of Lublin, 8 Solidarności St., 20-841 Lublin, Poland
[7] St. Padre Pio Provincial Hospital in Przemyśl, 18 Monte Cassino St., 37-700 Przemyśl, Poland
* Correspondence: daniel.piotr.zalewski@gmail.com (D.Z.); anna.bogucka-kocka@umlub.edu.pl (A.B.-K.); Tel.: +48-81-448-7236 (D.Z.); +48-81-448-7234 (A.B.-K.)

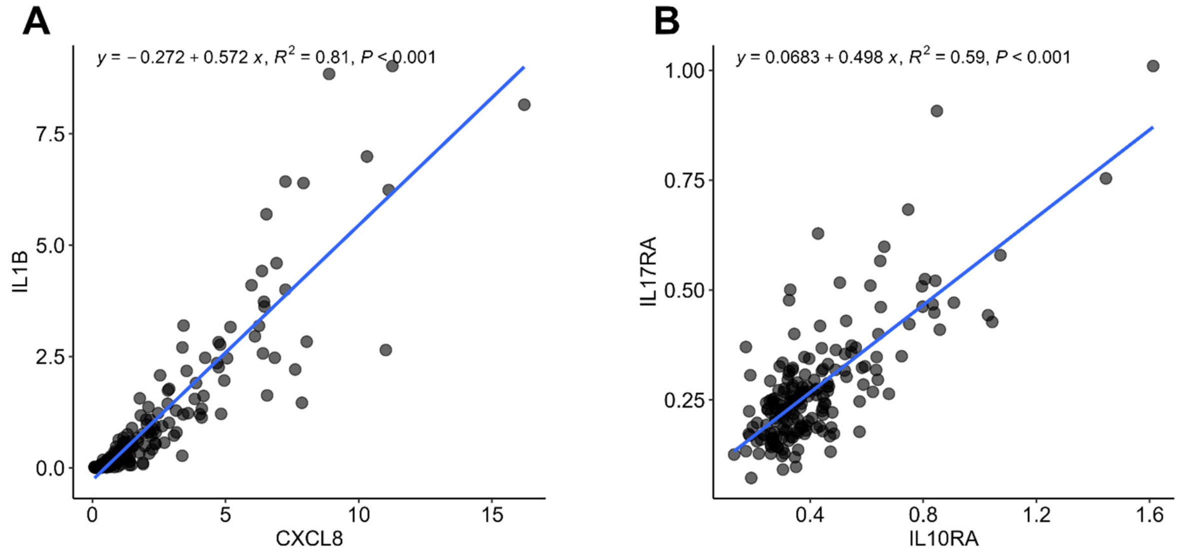

Figure S1. The most significant correlations found among the selected biomarker candidates. Trend lines and linear regression parameters were obtained using lm function in R.

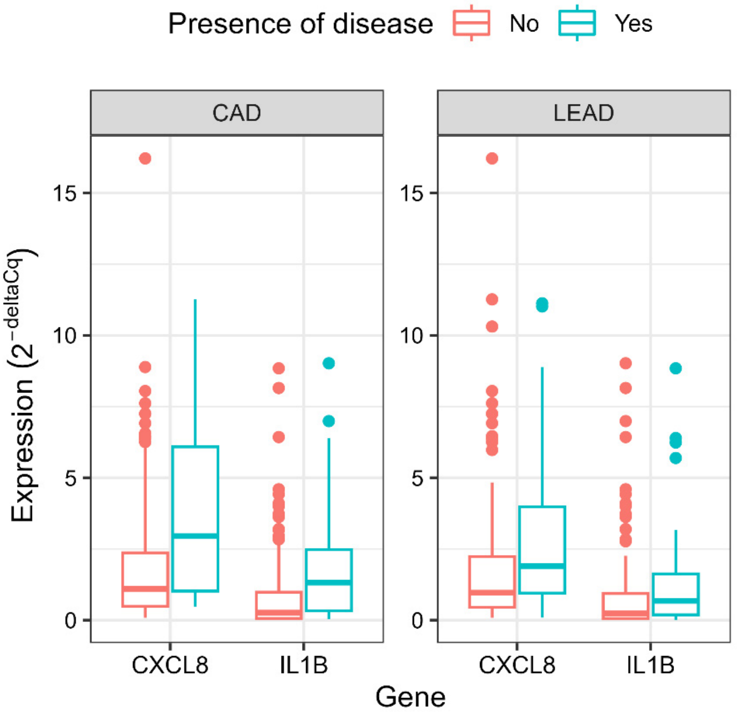

Figure S2. Distributions of *CXCL8* and *IL1B* expression levels in individuals with and without coronary artery disease (CAD) and lower extremity artery disease (LEAD). Whiskers reach the most distant point in the doubled interquartile range, boxes range between the 25% and 75% quartiles, and horizontal lines inside boxes mark the median values.

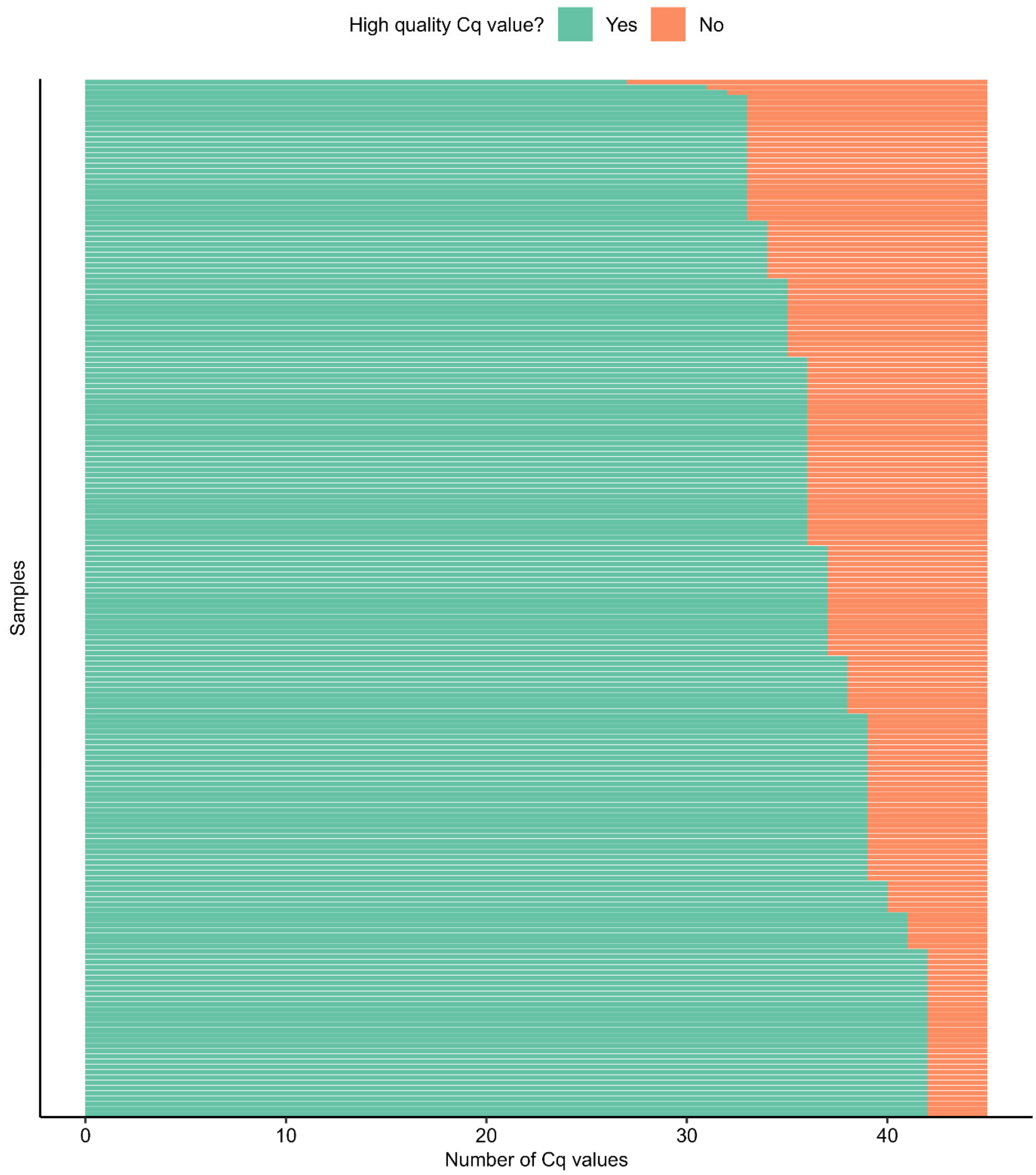

Figure S3. The amounts of genes with filtered and retained Cq values across analyzed samples.

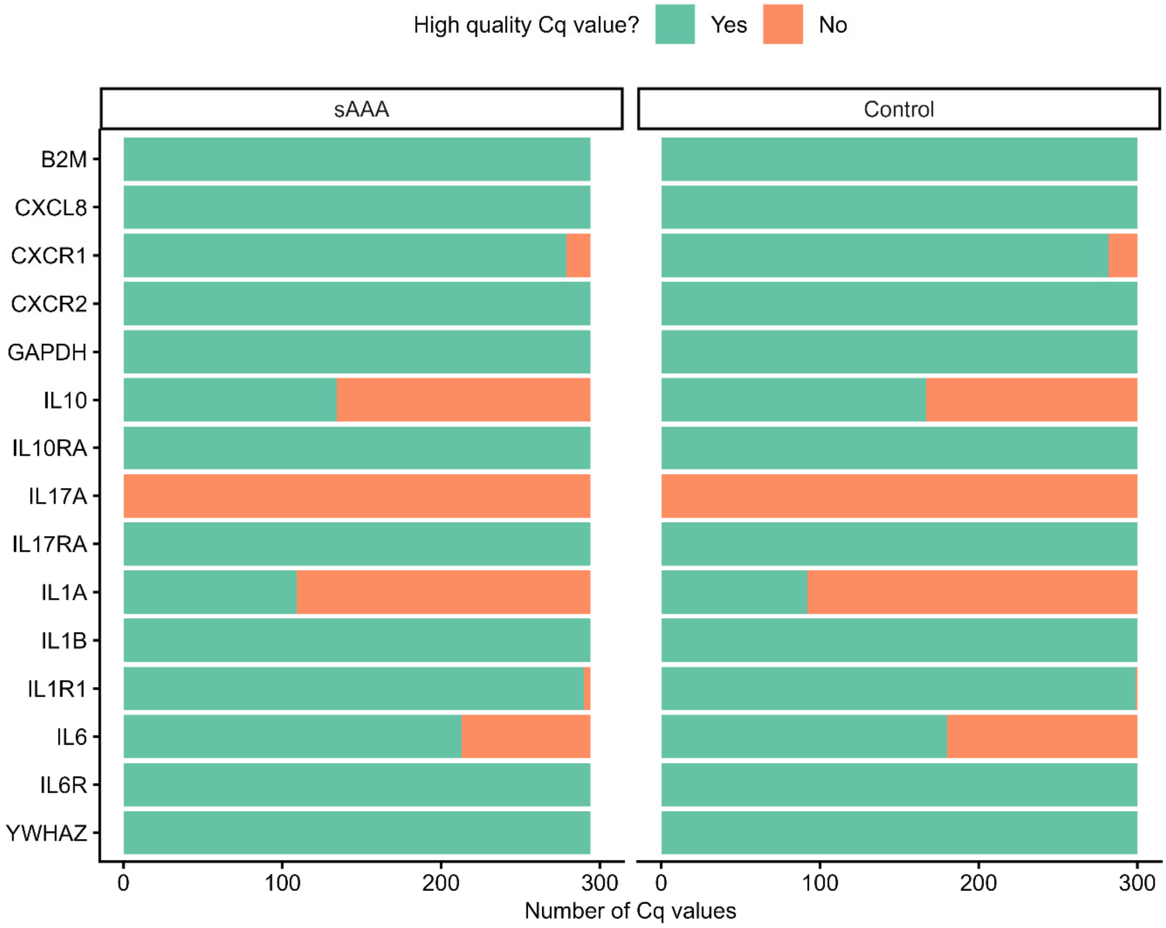

Figure S4. The amounts of samples (each in triplicates) with filtered and retained Ct values across analyzed genes (including endogenous control genes).

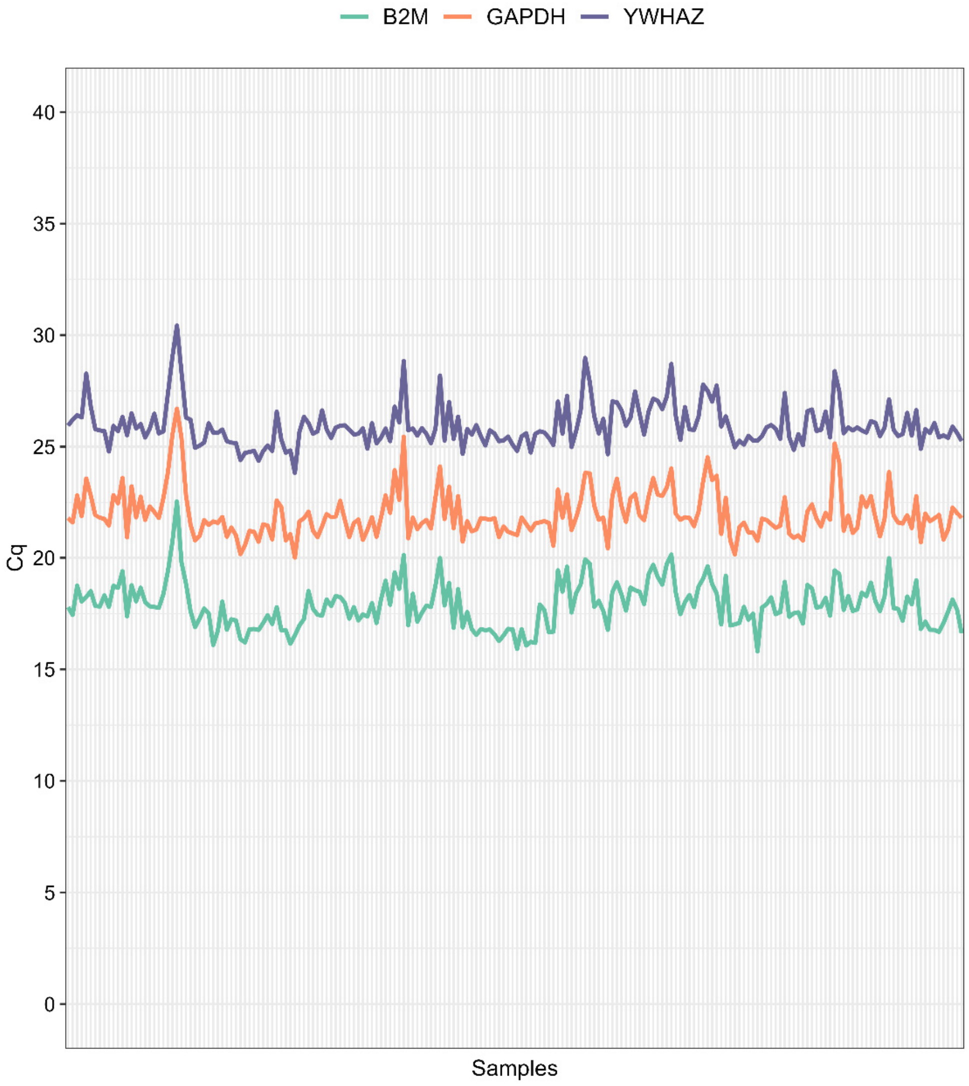

Figure S5. Raw Cq values obtained for three endogenous control genes used in the study as a potential data normalizators.

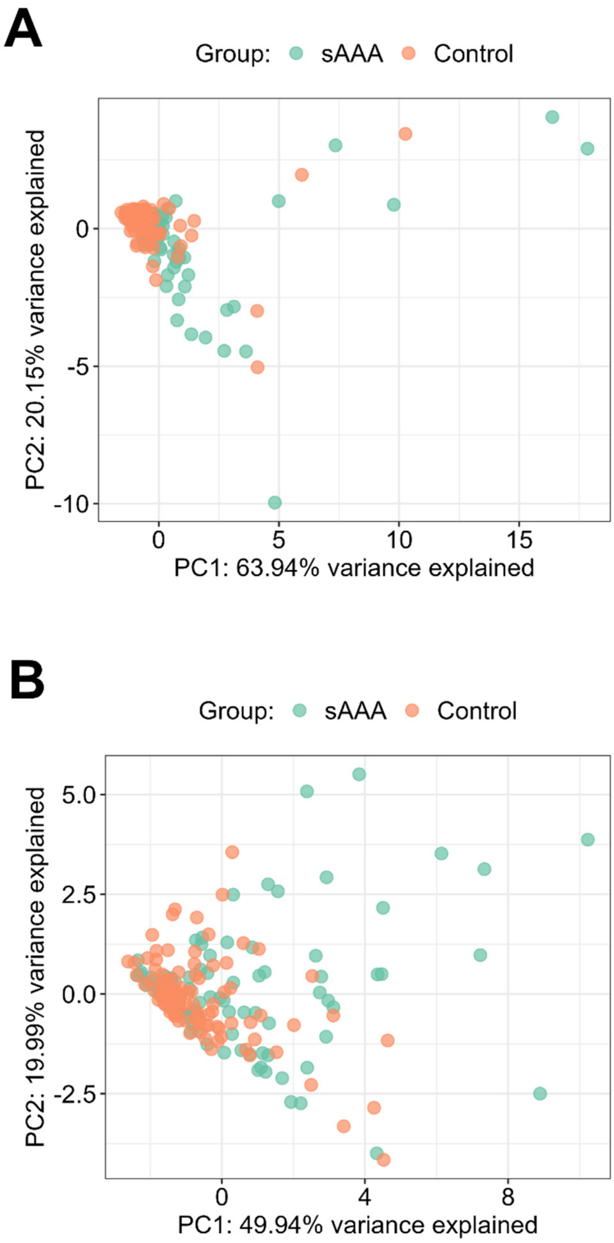

Figure S6. Spatial arrangement of samples using PCA components of normalized Cq data (A) before and (B) after outlier samples exclusion.

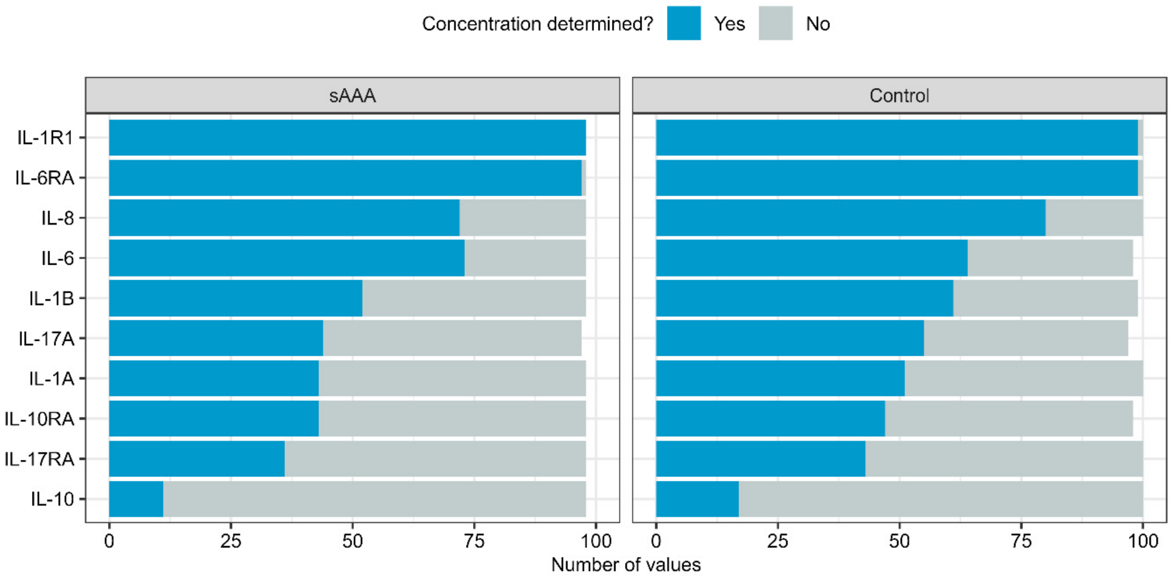

Figure S7. The amounts of samples with protein concentrations determined or not (above or below limit of detection).

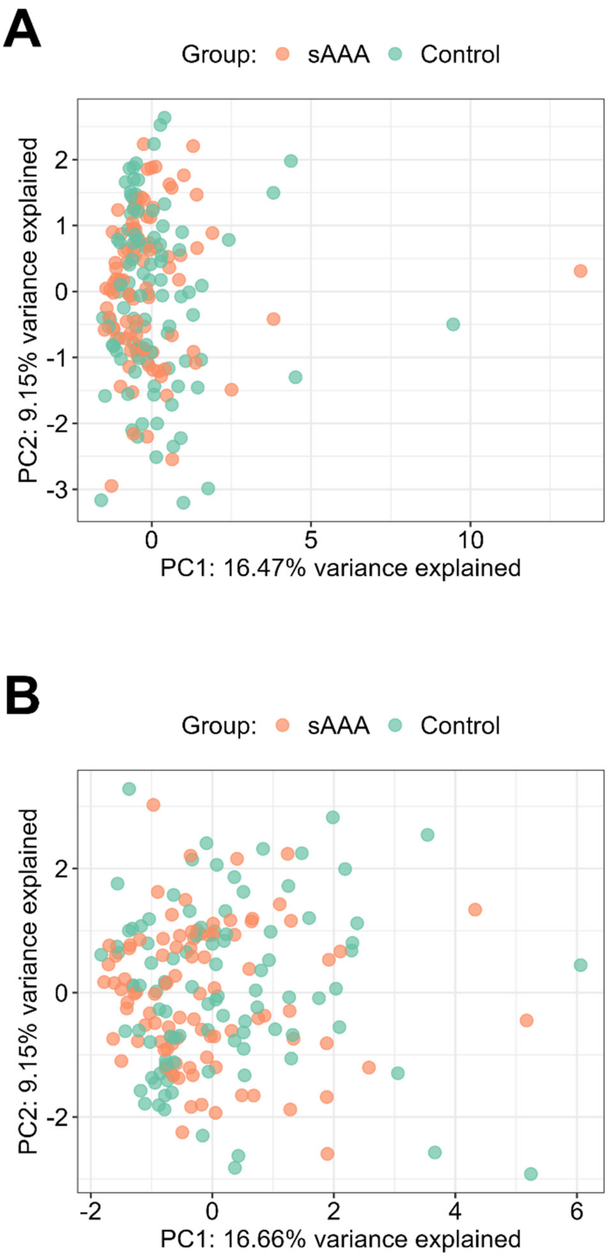

Figure S8. Spatial arrangement of samples using PCA components of protein plasma levels before (A) and after (B) outlier samples exclusion.
